# Out of sight but not out of harm’s way: Human disturbance reduces reproductive success of a cavity-nesting seabird

**DOI:** 10.1016/j.biocon.2014.03.020

**Published:** 2014-06

**Authors:** Hannah Watson, Mark Bolton, Pat Monaghan

**Affiliations:** a Institute of Biodiversity, Animal Health and Comparative Medicine, University of Glasgow, Glasgow G12 8QQ, UK; b RSPB Centre for Conservation Science, UK Headquarters, The Lodge, Sandy, Beds SG19 2DL, UK

**Keywords:** Tourism, Productivity, Reproductive behaviour, Seabird, Storm petrel, *Hydrobates pelagicus*, Visitor management, Recreational disturbance, Shetland

## Abstract

While negative effects of human disturbance on animals living above the ground have been widely reported, few studies have considered effects on animals occupying cavities or burrows underground. It is generally assumed that, in the absence of direct visual contact, such species are less vulnerable to disturbance. Seabird colonies can support large populations of burrow- and cavity-nesting species and attract increasing numbers of tourists. We investigated the potential effects of recreational disturbance on the reproductive behaviour of the European storm petrel *Hydrobates pelagicus*, a nocturnally-active cavity-nesting seabird. Reproductive phenology and outcome of nests subject to high and low levels of visitor pressure were recorded in two consecutive years. Hatching success did not differ between disturbance levels, but overall nestling mortality was significantly higher in areas exposed to high visitor pressure. Although visitor numbers were consistent throughout the season, the magnitude and rate of a seasonal decline in productivity were significantly greater in nests subject to high disturbance. This study presents good evidence that, even when humans do not pose a direct mortality risk, animals may perceive them as a predation risk. This has implications for the conservation and management of a diverse range of burrow- and cavity-dwelling animals. Despite this reduction in individual fitness, overall colony productivity was reduced by ⩽1.6% compared with that expected in the absence of visitors. While the colony-level consequences at the site in question may be considered minor, conservation managers must evaluate the trade-off between potential costs and benefits of public access on a site- and species-specific basis.

## 1 Introduction

Public access to wildlife is vital for generating support for biodiversity conservation, yet it is widely recognised that human disturbance can have negative effects on wildlife (reviewed by Boyle and Samson, 1985; Carney et al., 1999). Reported effects of human disturbance are varied and include changes in demography (e.g. Bolduc and Guillemette, 2003), behaviour (e.g. Duchesne et al., 2000), physiology (e.g. MacArthur et al., 1982) and distribution (e.g. Gill et al., 2001a). Most studies have focused on diurnal animals that primarily live above ground and out in the open. While some authors have considered the potential effects of human disturbance on animals concealed in refuges such as burrows, dens or cavities (Blackmer et al., 2004; Linnell et al., 2000; Magle et al., 2005; Steiner and Leatherman, 1981; Tempel and Gutiérrez, 2003), it is generally assumed that, by remaining out of sight, these animals are less vulnerable to the presence of human activities above ground (Burger, 1981; Lunn et al., 2004; though see Ellenberg et al., 2006). However, even when humans do not pose a direct mortality risk, animals may still perceive them as a predation risk and respond accordingly (Frid and Dill, 2002). While some animals may be able to relocate to an undisturbed location, in response to human disturbance, others may be forced to remain despite the disturbance (Gill et al., 2001b). The risks associated with leaving a refuge may be higher than staying and enduring a disturbance event. Furthermore, relocation may not be possible if breeding has already commenced or may be limited by energetic constraints or availability of alternative refuges. Even if breeding individuals can relocate, they may still suffer a reduction in reproductive output (Swenson et al., 1997).

Colonies of breeding seabirds can support a large number of burrow- and cavity-nesting species and are attracting increasing numbers of visitors. Seabirds are among the most globally-threatened groups of birds and human disturbance has been identified as one of the main threats they are facing (Croxall et al., 2012). Increasing recreational demands at breeding colonies have led to studies quantifying the impacts on breeding seabirds, but these have mostly focused on surface-nesting species (e.g. Beale and Monaghan, 2004). A few studies have examined the effects of investigator disturbance on burrowing seabirds and produced varied results: a reduction in hatching success was reported in response to nest-monitoring (e.g. puffins, Pierce and Simons, 1986) and handling of adults (e.g. storm petrels, Blackmer et al., 2004; e.g. shearwaters, Carey, 2011), while other studies found no effect of handling on breeding success (e.g. shearwaters, O’Dwyer et al., 2006). One study showed that merely the daily presence of a single researcher within a colony resulted in reduced fledging mass of young Cassin’s auklets (Albores-Barajas et al., 2009).

The effects of recreational disturbance, however, may be far greater than those of scientific investigators (Giese, 1996). While typically less invasive than scientific research, recreational visits are usually longer, less closely regulated and involve more people. A handful of studies have examined the effects of recreational activities on burrowing penguins. Reported effects include elevated corticosterone stress responses (Walker et al., 2005) and increased heart rate (Ellenberg et al., 2006), while another study showed no effects of tourism on breeding success (Yorio and Boersma, 1992). Rapid habituation to recreational disturbance was demonstrated in adults of the burrowing Magellanic penguin (Walker et al., 2006), but not in chicks (Walker et al., 2005). Unlike many other burrowing seabirds, however, penguins are active at the colony during the day and may come into visual contact with humans. There is clearly a need for conservation managers to quantify the effects of recreational activities and weigh up the costs and benefits of human access, but the information required to assess these impacts is rarely available (Gill, 2007).

In the present study, we investigated the effects of diurnal human disturbance associated with tourism on the reproductive performance of the European storm petrel *Hydrobates pelagicus*, a small seabird that nests in cavities and is strictly nocturnally-active at breeding colonies. Due to protracted development periods, incubating adults and chicks are present within the confines of the underground nest – and potentially vulnerable to disturbance - for extended periods. Although storm petrels remain out of visual contact with humans, they are exposed to odours, noise and vibrations associated with human activity close to or directly above their nests. We examined whether pairs nesting in areas exposed to high recreational disturbance differed in reproductive timing or outcome from those nesting in areas with little visitor activity. Reductions in breeding success have been widely reported in response to human disturbance. Birds may spend less time incubating their eggs, in response to disturbance (Verhulst et al., 2001; Wiegmann and Baylis, 1995), which may result in an extension to the incubation period and subsequent reduction in the likelihood of hatching (Chaurand and Weimerskirch, 1994). Reduced time spent brooding (Burger, 1981) or provisioning (Fernández and Azkona, 1993; Verhulst et al., 2001) young may increase offspring mortality. Alternatively, the breeding attempt may be abandoned altogether (Bolduc and Guillemette, 2003). We quantified the effects of disturbance on laying date, incubation period, hatching success, fledging success and overall productivity. While negative consequences for individual fitness are clearly important, we also quantified the magnitude of the observed effects at the colony level. Colony-scale effects will depend on both colony size and the proportion of breeding pairs affected by disturbance.

## 2 Methods

### 2.1 Study site and species

**Fig. 1:**
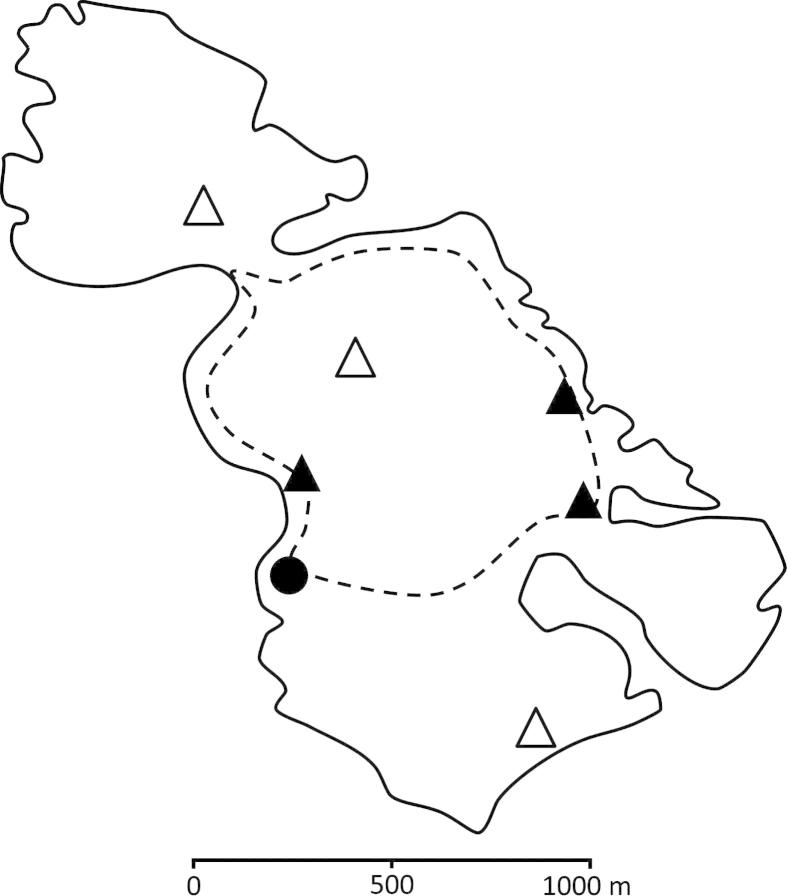
Map of study site illustrating the visitor path (dashed line), location of main archaeological interests including the Iron Age broch (closed circle), and location of study plots supporting storm petrel nests in areas subject to high (closed triangles) and low (open triangles) levels of recreational disturbance.

Mousa is a small (2 km^2^) island located in the Shetland archipelago, UK (60°0′N, 1°10′W). The site is managed as a nature reserve by the Royal Society for the Protection of Birds and is designated under the EU Birds Directive as a Special Protection Area, for which the storm petrel is a qualifying feature. The island comprises flat coastal grassland, surrounded by cliffs and boulder beaches. During the breeding season, Mousa receives 4000–5000 visitors during daylight hours, attracted by wildlife and archaeological interests. The island has remained uninhabited since 1853 and is not exposed to any regular sources of human disturbance outside of tourism during the summer months. The average number of diurnal visitors per day is consistent throughout the main part of the breeding season (June: 27.8 ± 3.1; July: 26.3 ± 3.7; August: 27.9 ± 3.1). Although access to the island is unrestricted, visitor management ensures that the majority of activity is restricted in space and time. A single daily ferry sailing provides access to the island and limits the duration of visits (usually <3 h). The provision of maps and information signs acts to concentrate activity within a 3.2 km circular route in the central portion of the island (Fig. 1); furthermore, the key points of interest are all situated along this trail, therefore there is little incentive to stray far from the path. While there is some tourism at night, comprising guided walks to observe storm petrels as they enter and leave the colony just after darkness, this is strictly controlled (no torches; duration *c*. 1.5 h), and restricted to a small area of the island and the early part of the breeding season.

On Mousa, storm petrels nest in crevices in dry stone walls, boulder beaches, loose rock scree, and abandoned buildings including a 2000-year-old Iron Age broch (dry stone tower). Like many other burrow- and cavity-nesting seabirds, storm petrels are only active within the colony at night. During the day, birds either remain in the underground nest, out of sight, or are foraging at sea. As a consequence of their nocturnal habits and the inconspicuousness of nests, diurnal visitors are generally unaware of their proximity to breeding storm petrels. Storm petrels prefer dark chambers and nests are usually at least 30 cm below ground and without a direct view to the exterior; therefore, there is no visual contact between diurnal visitors and storm petrels. Storm petrels do not use earth burrows at this site and therefore there are no risks concerning erosion or burrow collapse due to the presence of recreational activity. The storm petrel lays a single egg that is incubated by both parents for *c*. 40 d (Davis, 1957). The nestling is brooded for about a week, during which time one adult remains with the chick while the other is foraging (Mínguez and Oro, 2003). During the remainder of chick-rearing, the nestling remains alone in the underground nest, while both parents are foraging, returning most nights to feed the nestling; the chick fledges at 60–70 d (Davis, 1957). The island remains free from mice, rats and cats; the only mammalian predator present is the Eurasian otter *Lutra lutra*, which rarely predates on storm petrel nests.

Based on observations of visitor behaviour, we concluded that recreational activity predominates within a belt of 10 m either side of the marked path. For this reason, we considered nests located within 10 m of the path to be subject to ‘high’ levels of visitor pressure. Visitors rarely explore beyond this area and we adopted a conservative threshold distance of 150 m from the path, beyond which we considered nests to be subject to ‘low’ levels of visitor pressure. A total of 95 study nests were distributed among six ‘plots’, geographically spread across the island (Fig. 1). Three plots, encompassing 43 nests, were in areas subject to high disturbance (i.e. ⩽10 m of the path), while the other three plots, supporting 52 study nests, were in areas subject to low disturbance (i.e. >150 m from the path). Study nests were located in either walls or rock scree and both of these nesting habitats were equally represented within the high and low disturbance categories. Nesting densities were similar between high- and low-disturbance plots. There were no other differences in surrounding habitat or environmental conditions between plots. Only one of the high-disturbance plots was exposed to any nocturnal human disturbance (see above).

### 2.2 Effects on individual reproductive performance

In the consecutive breeding seasons of 2010 and 2011, 75 and 82 of the study nests, respectively, were occupied by a breeding pair of storm petrels. Nests were monitored to determine laying date, hatching date and breeding outcome. Nests were visually inspected most days from early June, allowing the determination of laying date with a maximum error of 3 d for at least 85% of breeding records. To minimise disturbance, nests were inspected briefly with the aid of a torch. Storm petrels are highly sensitive to disturbance induced by handling during incubation, so neither the egg nor incubating adults were removed from nests. In 2010, nests continued to be inspected regularly after laying to record nest attendance (not reported here). In 2011, once laying was confirmed, inspections ceased until 38 d after the earliest possible laying date - close to expected hatching. The average level of investigator disturbance experienced by nests was thus constant between disturbance levels within years, but higher in 2010 than in 2011.

Limited visibility at *c*. 15% nest sites prevented hatching dates from being accurately determined from visual checks. At these sites, the age of nestlings (and subsequently hatching date) was estimated from tarsus length. Up to *c.* 30 d the tarsus grows at a constant rate (Bolton, unpubl.) allowing age to be estimated from the linear regression of age on tarsus length of known-age chicks (2010: *r*^2^ = 0.94; 2011: *r*^2^ = 0.96). This enabled estimation of age to within 3 d of actual age in 94% of nestlings. Due to increasingly adverse weather conditions restricting access to the island late in the season, most nests were not followed through to fledging. The majority of chicks were between 50 and 60 d at the final check and all were >30 d, beyond which failure is unlikely and successful fledging can be confidently assumed (Davis, 1957). Of 116 chicks hatched over two years, just one died beyond 18 d. Nests were inspected at the beginning of the following season to provide additional confirmation of whether the chick fledged successfully or not. Nest predation is rare and obvious, since the only predator present capable of removing a chick from a nest is the otter, which always leaves clear signs of excavation.

### 2.3 Population-level effects

To quantify the effect of recreational disturbance at the population level, we first needed to quantify the proportion of the colony that is subject to high levels of visitor disturbance. Although the colony had recently been censused in 2008 (Bolton et al., 2010), this did not give separate estimates for each of the areas subject to high and low disturbance. Between 21 and 27 July 2012, we conducted a census of all potentially-suitable nesting habitat within 10 m of the visitor path. We employed the playback methodology, developed by Ratcliffe et al. (1998) and used in two previous whole-island surveys (Bolton et al., 2010; Ratcliffe, 1997), in which a male purr call is played at 1 m intervals along transects and the number of individual responses recorded. In addition to censusing wall, boulder and rock scree habitats (as per Ratcliffe, 1997), we also surveyed the Mousa broch, an Iron Age stone tower, which was not included in previous island surveys. The broch’s structure essentially consists of two 13 m-high concentric walls. For the purposes of the survey, each of the two walls were considered to consist of 13 individual metre-high “walls” situated one on top of another. The lowest two “walls” (i.e. from 0–1 m and 1–2 m above ground) and the uppermost “wall” (12–13 m) of both the external and internal walls of the broch were surveyed following the methodology described for wall habitats (see Ratcliffe, 1997).

Since not all birds will respond to playback, it was necessary to calibrate the number of responses recorded (as per Ratcliffe et al., 1998). Playback was conducted at 55 sites known to be occupied by a breeding pair. The response rate was calculated as the proportion of occupied sites from which a response was elicited on a single day. The response density for each surveyed area was subsequently adjusted for the response rate and multiplied by the total length (walls) or total area (rocks or boulders) to give an estimate of apparently occupied sites (AOSs). Summing the estimated number of AOSs for all surveyed areas gave us the total number of AOSs subject to high levels of visitor disturbance. Subtracting the number of high-disturbance AOSs from the most recent colony estimate of 11781 AOSs (Bolton et al., 2010) provided us with an estimated number of AOSs subject to low visitor disturbance. Subsequently, using productivity rates from high- and low-disturbance areas determined from the present study, we predicted the number of offspring fledged from the colony in the presence and absence of recreational disturbance in 2012. We express the colony-level consequences associated with recreational disturbance in terms of the predicted annual reduction in colony productivity due to tourism.

### 2.4 Statistical analysis

All statistical analyses were performed in R 3.0.0 (R Core Team, 2013). Linear mixed models with a normal error structure were employed to examine variation in laying date in relation to disturbance using the lme4 package (Bates et al., 2013). We considered the random effects of nest identity and nest identity nested within plot. First, the optimal random effects structure was determined by performing likelihood ratio tests (LRTs) on nested models fitted by restricted maximum likelihood (REML), with a saturated fixed component and different random effects structures. The variance associated with the random effect of plot in all models was close to zero and did not significantly improve the model fit, so only nest identity was retained as a random effect. The saturated model included the fixed effects of disturbance and year and the interaction between disturbance and year. The optimal fixed effects structure was obtained by stepwise deletion, sequentially removing non-significant parameters (*P* > 0.05) from models fitted by maximum likelihood (ML) estimation. The significance of model parameters was estimated by comparison to a probability distribution obtained from 10,000 Markov Chain Monte Carlo simulations using pvals.fnc() in languageR, which does not require the estimation of degrees of freedom. Results are presented from the minimum adequate model fitted by REML. Differences in incubation period (the number of days from laying to successful hatching) between nests subject to high and low visitor pressure were examined using Wilcoxon rank-sum tests for each year separately. Unless stated otherwise, all results are presented as means ± SE.

Since complete nest histories were available for all nests, reproductive success was analysed using a binary (0/1) response variable. Generalised linear mixed models (GLMMs) with a binomial error structure and logit link function were fitted to data on (i) overall productivity, (ii) hatching success and (iii) fledging success. Again, the variance associated with the random effect of plot was close to zero and only the random effect of nest identity was retained in models. Saturated models included the fixed effects of disturbance (high or low), laying date (covariate), year (2010 or 2011) and nest type (wall or scree). We also considered relevant two-way interactions to control for seasonal (laying date × disturbance) and inter-annual (year × disturbance) variation in the effect of disturbance on the response variable and inter-annual variation in the effect of laying date on the response variable (year × laying date). The optimal fixed effects structure was attained by a stepwise deletion process, in which each term was removed separately and LRTs were performed between each of the reduced models and the fuller model. The criteria for removal of a term was a log-likelihood ratio *P*-value > 0.05. MCMC sampling is not yet implemented for generalised linear mixed models in lme4; therefore, the significance of parameter estimates from minimum adequate models was estimated using Wald *z*-tests. The predictive accuracy of models was assessed using Receiver Operating Characteristic plots fitted using the ROCR package (Sing et al., 2005). The resulting area under the curve (AUC) offers a measure of predictive performance; a value of 1.0 indicates a perfect model, while a value of 0.5 indicates that a model performs no better than random. AUC values are reported for final models.

## 3 Results

### 3.1 Visitor pressure and reproductive performance

**Fig. 2:**
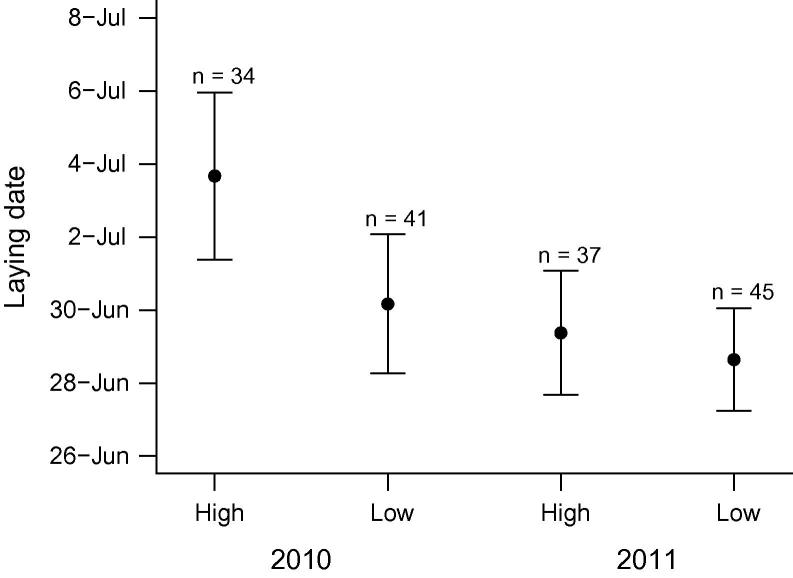
Laying date of storm petrels nesting in areas subject to high and low recreational disturbance in 2010 and 2011 at Mousa, Shetland (all *P* > 0.1). Means ± SE are presented with corresponding sample sizes.

A summary of reproductive performance of storm petrels breeding in areas subject to high and low levels of recreational disturbance in the two study years is presented in Table 1. Egg-laying began in early June and continued through to early August in both years. There was no effect of visitor pressure (*t* = 1.00, *P* = 0.264) or year (*t* = −2.24, *P* = 0.113) on laying date (Fig. 2) and the effect of disturbance did not vary between years (disturbance (low) × year: *t* = 0.85, *P* = 0.452). The length of the incubation period was unaffected by visitor pressure in either 2010 (median and range: high: 40 d, 39–43 d; low: 40 d, 39–42 d; *W* = 397, *P* = 0.565, *n* = 55) or 2011 (median and range: high: 42 d, 39–66 d; low: 42 d, 39–69 d; *W* = 451, *P* = 0.697, *n* = 62). Failure during the nestling stage was recorded in 12 and 25 nests in 2010 and 2011 respectively. Excluding one chick that died at 56 d, the majority of mortalities (78%) occurred at ⩽8 d (median: 6 d; range: 1–18 d; *n* = 36).

**Fig. 3:**
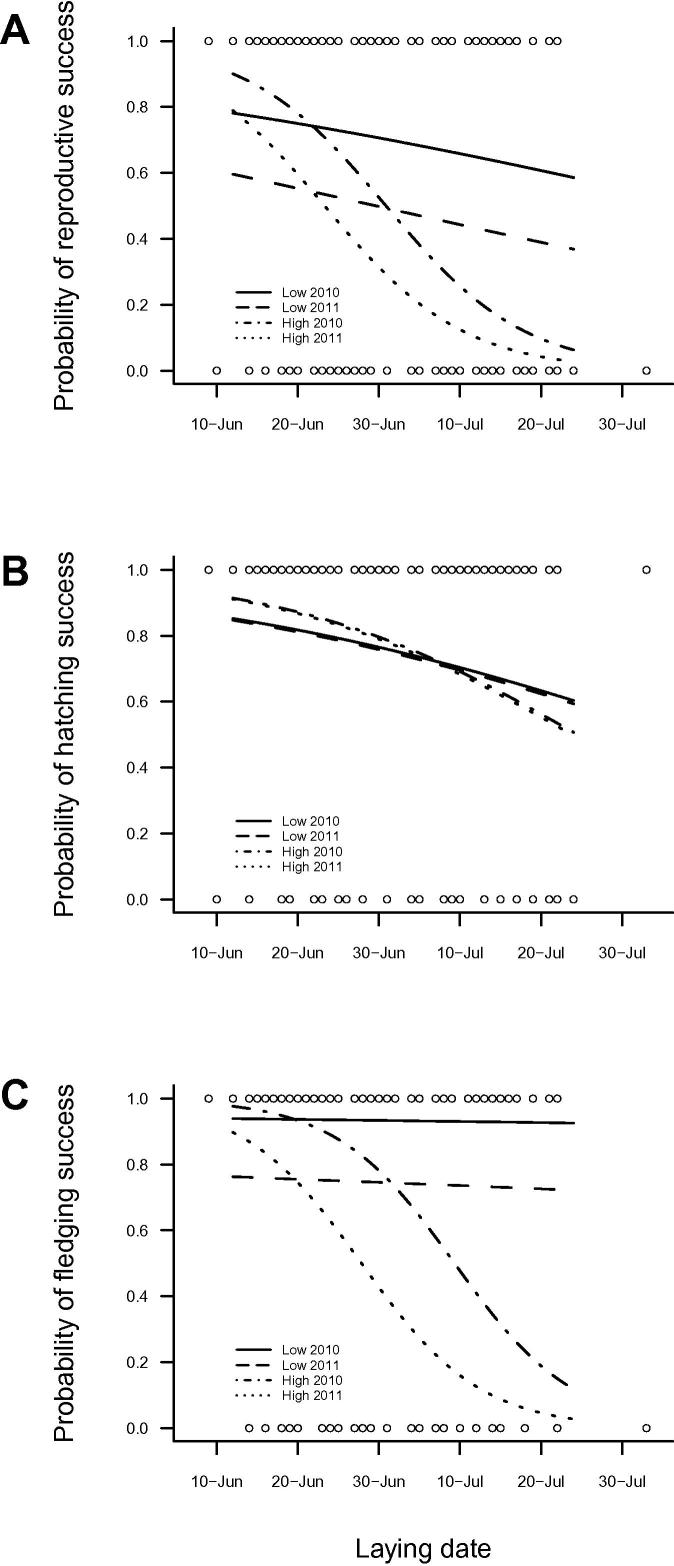
Seasonal trend in the probability of (A) reproductive success (*n* = 157), (B) hatching success (*n* = 157), and (C) fledging success (*n* = 117) of storm petrels nesting in areas subject to high and low recreational disturbance in a ‘good’ (2010) and a ‘poor’ (2011) year for overall colony productivity. Lines represent predictions from GLMMs fitted within the range of observed values (open circles). See Table 2.

The likelihood of reproductive success was significantly lower in nests exposed to high levels of visitor disturbance compared with low-disturbance nests (Tables 1 and 2; *P* = 0.021). However, a significant interaction between laying date and disturbance (Table 2; *P* = 0.016) confirmed different patterns of seasonal decline between high- and low-disturbance nests. The rate and magnitude of seasonal decline in probability of successful breeding was greater in areas subject to high levels of visitor pressure (Table 2, Fig. 3A). While the likelihood of successful hatching decreased significantly with increasing laying date (Table 2; *P* = 0.017), there was no effect of disturbance (*z* = −0.29, *P* = 0.771) or year (*z* = −0.06, *P* = 0.949) on hatching success (Fig. 3B). In contrast, a significant interaction between laying date and disturbance (Table 2, Fig. 3C; *P* = 0.027) revealed a marked seasonal decline in probability of fledging success in high-disturbance nests, while there was very little seasonal change in fledging success in nests subject to low levels of recreational pressure. Both the likelihoods of reproductive success (Table 2, Fig. 3A; *P* = 0.020) and fledging success (Table 2, Fig. 3C; *P* = 0.011) were significantly lower in 2011 compared with 2010 across all nests, yet the effect of disturbance persisted. There was no significant difference in productivity (*z* = −1.63, *P* = 0.102), hatching success (*z* = −0.72, *P* = 0.473) or fledging success (*z* = −1.73, *P* = 0.084) between wall and scree nesting habitats.

### 3.2 Colony-level effects

The playback survey elicited a total of 66 responses to playback from 1076 survey points. Of 55 calibration sites known to be occupied by a breeding pair, a response to playback was elicited from 12 sites, giving a response rate of 0.218. This is comparable to response rates determined in previous censuses (Ratcliffe, 1997; Ratcliffe et al., 1998; Bolton et al., 2010). The mean response densities, adjusted for the response rate, were 0.41 AOSs m^−1^ for walls (including the broch) and 0.18 AOSs m^−2^ in natural habitats. When multiplied by the total area of suitable habitat, this yielded estimates of 330 AOSs and 197 AOSs in walls and natural habitats respectively, giving an estimated total of 527 AOSs located within 10 m of the visitor path and subject to high visitor pressure in 2012. Based on a predicted population size of 11,781 breeding pairs (see Bolton et al., 2010), this equates to 4.5% of the colony being subject to high levels of recreational disturbance in 2012 and, therefore, reduced annual productivity, compared with the remainder of the population, which is subject to low levels of disturbance. Using productivity estimates from both 2010 and 2011, between 70 and 128 fewer chicks were estimated to have fledged from the colony in 2012 compared with a hypothetical scenario where the whole population is subject to low disturbance (Table 3). This represents a reduction in overall colony productivity ranging from 1.2–1.6%.

## 4 Discussion

Despite nesting underground and out of sight, reduced reproductive success of a cavity-nesting nocturnal bird was associated with diurnal human disturbance above ground due to tourism. There were no differences in incubation period or hatching success between high- and low-disturbance nests, suggesting birds exposed to repeatedly high levels of visitor disturbance can maintain incubation effort, but early nestling mortality was significantly higher. We showed that the relationship was spatially and temporally stable, as demonstrated by repeatable results between the different plots within each disturbance treatment and between two consecutive seasons, respectively. The relationship between disturbance and breeding success persisted in both a ‘good’ and a ‘poor’ year for overall colony productivity and therefore presumably under very different environmental conditions. The colony has been exposed to tourism at this level for a number of years, suggesting that, if any habituation to human presence has occurred, it is not sufficient to offset negative impacts. There was no evidence to support alternative explanations for the observed reduction in breeding success. Availability of nesting habitat is not limited at the study site and different nesting habitat types (wall and rock scree) were equally represented within the two disturbance levels. There were no site-specific differences between high- and low-disturbance plots besides the level of visitor activity. Investigator pressure was the same at high- and low-disturbance nests within years and, in fact, the probability of reproductive success was higher in 2010 when investigator disturbance was also higher. Furthermore, breeding success was comparable to that recorded by low-intensity annual monitoring in the same years in low-disturbance areas (0.50 and 0.56, respectively, Okill, unpubl.), suggesting that investigator disturbance did not significantly influence breeding success.

An alternative explanation for the differences in reproductive success observed between disturbed and undisturbed areas could be variation in individual quality which could potentially arise through perceived differences in habitat quality according to human disturbance pressures. In the absence of an experimental manipulation of visitor activity (which was not possible at this site), we cannot wholly discount this possibility. However, if disturbed areas did support birds of lower quality, it would be expected that birds in high-disturbance areas would breed later in the season (Verhulst et al., 1995). In fact, we found no difference in the timing of breeding between disturbance levels, which suggests that the differences observed in reproductive success between high- and low-disturbance areas were not linked to age, experience or condition of adults (Mills, 1973). Prior to laying, storm petrels spend little time, if any, at the colony during daylight and are therefore limited in their ability to assess cues related to diurnal human disturbance. We thus expect individuals to be randomly distributed, with respect to individual quality, between disturbed and undisturbed areas.

The cause of reproductive failure in response to disturbance is unknown, though there was no evidence for predation or damage to nests. Breeding failure due to human disturbance is often attributed to birds fleeing the nest, leaving the contents vulnerable to predation or at the mercy of the weather (e.g. Bolduc and Guillemette, 2003). Storm petrels were, however, never observed to flee from the nest during the day; the risks associated with leaving the nest are likely to be far greater than staying and enduring a disturbance event. The majority of nestling mortality occurred shortly after hatching; the determination of cause of death would have required a high level of investigator disturbance at the nest. It is therefore not possible to determine whether mortality was driven by abandonment, poor parental care or due to an intrinsic characteristic of the chick itself. There are thus several potential routes by which disturbance could influence nestling survival, either via direct effects on the chick itself, for example via increased physiological stress or elevated energetic demands, or via indirect effects on parental care during incubation and/or the early chick-rearing period. According to life-history theory, animals are expected to adjust current reproductive investment relative to the costs of future survival and reproduction (Stearns, 1992). The trade-off between current reproduction and survival is likely to be more marked in long-lived animals, which are expected to favour the maintenance of their own body condition over that of their young (Drent and Daan, 1980). Breeding seabirds may thus be particularly responsive to disturbance by humans and more likely to reduce parental effort compared with shorter-lived animals. Although there were no differences in the length of the incubation period or hatching success between high- and low-disturbance nests, it has previously been shown that the prenatal environment can exert constraints on postnatal development even in the absence of effects on incubation period or hatching success (Nilsson et al., 2008).

Previous studies have assumed that visual contact is necessary to invoke a disturbance event (Lunn et al., 2004; Yorio and Boersma, 1992) and, therefore, refuge-dwelling animals are less vulnerable to the effects of human disturbance. However, it was shown that a human passing a nest-burrow, out of visual contact, was sufficient to elicit an elevation in heart rate in incubating Humboldt penguins (Ellenberg et al., 2006). The present study supports the work of Ellenberg et al. (2006), demonstrating that negative effects of human activities can occur even when animals do not see the intruder and appear to be protected from impact. This study presents good evidence that, even when humans do not pose a direct mortality risk, they may still be perceived as a predation risk. The suggestion that disturbance can affect animals in refuges via routes other than visual contact, due to noise, vibrations or even odours, has implications for the conservation and management of a wide range of species that utilise refuges.

Most studies of the impacts of recreational disturbance have focused on individual responses, with little consideration for consequences at the population level (though see Mallord et al., 2007; Patthey et al., 2008). While the fitness consequences of human disturbance are clearly important at the individual level, whether these effects result in population-level impacts will depend on the scale at which the disturbance occurs (Gill, 2007). By quantifying the effects at the colony level as well as the individual level, we were able to demonstrate that, at the current level of provision of visitor access at the study site, less than 5% of the population are vulnerable to the effects of recreational disturbance. The storm petrel colony at Mousa has more than doubled in size since 1996 (Bolton et al., 2010), in the presence of tourism, and therefore, under the present visitor management scenario, it is not expected that visitor activity will prevent further growth of this colony. However, even a small reduction in population growth rate could have significant implications for seabirds, which are characterised by low fecundity and delayed maturation. Population-level consequences may be more dramatic in other situations, particularly if the population is under additional pressure, for example due to non-native predators or being located at the edge of the species’ range.

Conservation management always requires an evaluation of the trade-off between the costs and benefits of public access; in the present case, a small reduction in colony productivity may be accepted in favour of the potential benefits of tourism. This study demonstrates that the potential impacts of visitor activity can be minimised through a combination of a number of management measures including the safeguarding of core areas as refuges, the use of suitably-located and well-marked trails and viewing points, and limited times of access. Recreational users may be unaware of the presence of animals in cavities or burrows and, in contrast to investigators, are less likely to adjust their behaviour accordingly to minimise impacts. It is therefore essential to provide clear guidance to visitors to facilitate low-impact recreational access. Small changes in visitor management, however, have the potential to significantly alter the scale and magnitude of effects. This highlights the importance for conservation managers to have access to sufficient information to enable them to quantify potential impacts at both the individual and population level and use this to effectively inform management practices. Managers should explore both traditional and novel opportunities for offsetting restrictions on access by offering visitors an enhanced experience of their visit; to name but a few suggestions, this could include provision of high-quality interpretation boards, remote viewing facilities and interactive web-based resources that visitors can access following their visit.

## 5 Conclusions

Increasing interest in nature-based tourism is expected to lead to increased pressures on coastal and marine habitats and the seabird colonies they support. Burrowing seabirds belonging to the Procellariiformes (shearwaters and petrels), Alcidae (auks) and Sphenisciformes (penguins) are already facing extensive threats at breeding sites from loss and degradation of habitat as a result of human interference and introduction of mammalian predators, as well as impacts at sea, such as mortality in long-line fisheries (Croxall et al., 2012). The effects of tourism could exacerbate the population declines that are widely reported amongst seabirds. Although further research is required to determine the proximate cause of failure associated with disturbance, the study presents good evidence that, even in the absence of visual contact, the presence of human activity above ground can have negative effects on animals remaining out of sight in refuges. Humans may be perceived as a predation risk and stimulate appropriate anti-predator responses, even in the absence of a direct mortality risk. Negative consequences for individual fitness, however, do not necessarily translate to population-level effects and thus it is critical that conservation managers have the necessary information to evaluate impacts at both the individual and population level. The relative costs and benefits of public access should be evaluated on a site- and species-specific basis. While the results have most immediate relevance to the conservation and management of seabird colonies across the world, they also highlight the potential for impacts of recreational disturbance on a wider range of species utilising refuges. Conservation managers should consider the potential for impacts on less obvious animals and it should not be assumed that animals remaining out of sight are out of harm’s way.

## Figures and Tables

**Table 1:** Summary of reproductive performance of storm petrels nesting in areas subject to high and low recreational disturbance in 2010 and 2011 at Mousa, Shetland.

	*n*	Productivity	Hatching success	Fledging success
*2010*				
High disturbance	34	0.44	0.71	0.63
Low disturbance	41	0.68	0.76	0.90

*2011*				
High disturbance	37	0.38	0.78	0.48
Low disturbance	45	0.51	0.73	0.70

**Table 2:** Summaries of minimum adequate GLMMs fitted to data on productivity (*n* = 157), hatching success (*n* = 157) and fledging success (*n* = 117) of storm petrels nesting in areas subject to high and low levels of recreational disturbance in 2010 and 2011^a^^,^^b^.

Dependent variable	Fixed effect	Estimate ± SE	*z*-Value	*P*
Productivity	Laying date	−0.117 ± 0.03	−3.65	<0.001
	Disturbance (low)	−16.38 ± 7.15	−2.29	0.021
	Year (2011)	−0.885 ± 0.38	−2.33	0.020
	Laying date × disturbance (low)	0.095 ± 0.04	2.40	0.016

Hatching success	Laying date	−0.042 ± 0.02	−2.43	0.017

Fledging success	Laying date	−0.137 ± 0.04	−3.16	0.002
	Disturbance (low)	−22.45 ± 10.8	−2.09	0.037
	Year (2011)	−1.569 ± 0.62	−2.53	0.011
	Laying date × disturbance (low)	0.132 ± 0.06	2.21	0.027

a All models included the random effect of nest identity.

b The predictive performance of models was assessed using Receiver Operating Characteristic plots, which yielded values of 0.88, 0.81 and 0.94 for the area under the curve (AUC) for each respective model.

**Table 3:** Estimated productivity of the storm petrel colony at Mousa in the presence and absence of visitors based on an estimated population size of 11,781 breeding pairs (Bolton et al., 2010), of which 527 pairs nest in areas currently subject to high levels of visitor disturbance. Estimates are generated based on a ‘good’ and ‘poor’ scenario of colony productivity, using productivity rates from 2010 and 2011 respectively (see Table 1).

	Number of chicks fledged		
	High-disturbance areas		Low-disturbance areas
	With visitors	Without visitors	With/without visitors
2010	232	360	7686
2011	199	269	5751
